# cAMP Catalyzing Phosphodiesterases Control Cholinergic Muscular Activity But Their Inhibition Does Not Enhance 5-HT_4_ Receptor-Mediated Facilitation of Cholinergic Contractions in the Murine Gastrointestinal Tract

**DOI:** 10.3389/fphar.2018.00171

**Published:** 2018-03-08

**Authors:** Vicky Pauwelyn, Romain A. Lefebvre

**Affiliations:** Department of Pharmacology, Heymans Institute, Ghent University, Ghent, Belgium

**Keywords:** 5-HT_4_ receptor, cholinergic neurotransmission, gastrointestinal tract, mouse, phosphodiesterase, prucalopride, smooth muscle

## Abstract

**Background:** As the signal transduction of 5-HT_4_ receptors on cholinergic neurons innervating smooth muscle is controlled by phosphodiesterase (PDE) 4 in porcine stomach and colon, and human large intestine, the *in vivo* gastroprokinetic effects of a 5-HT_4_ receptor agonist might be enhanced by combination with a selective PDE4 inhibitor. The presence of 5-HT_4_ receptors on cholinergic neurons towards murine gastrointestinal circular muscle was recently shown. If the control of this receptor pathway by PDE4 is also present in mice, this might be a good model for *in vivo* testing of the combination therapy. Therefore this study investigates the role of cAMP catalyzing PDEs in smooth muscle cell activity and in the intraneuronal signal transduction of the 5-HT_4_ receptors in the gastrointestinal tract of C57Bl/6J mice.

**Methods:** In circular smooth muscle strips from murine fundus, jejunum, and colon, submaximal cholinergic contractions were induced by either electrical field stimulation (EFS) or by carbachol (muscarinic receptor agonist). The influence of the PDE inhibitors IBMX (non-selective), vinpocetine (PDE1), EHNA (PDE2), cilostamide (PDE3), and rolipram (PDE4) was tested on these contractions and on the facilitating effect of a submaximal concentration of prucalopride (5-HT_4_ receptor agonist) on EFS-induced contractions.

**Results:** In the three gastrointestinal regions, IBMX and cilostamide concentration-dependently decreased carbachol- as well as EFS-induced contractions. Some inhibitory effect was also observed with rolipram. In the fundus a non-significant trend for an enhancement of the facilitating effect of prucalopride on EFS-induced contractions was observed with IBMX, but none of the selective PDE inhibitors enhanced the facilitating effect of prucalopride in fundus, jejunum or colon.

**Conclusion:** In analogy with the porcine gastrointestinal tract, in murine fundus, jejunum, and colon circular smooth muscle PDE3 is the main regulator of the cAMP turnover, with some contribution of PDE4. In contrast to the porcine gastrointestinal tract, the *in vitro* facilitation of electrically induced cholinergic contractions by 5-HT_4_ receptor stimulation could not be enhanced by specific PDE inhibition. The C57Bl/6J murine model is thus not suitable for *in vivo* testing of a 5-HT_4_ receptor agonist combined with a selective PDE4 inhibitor.

## Introduction

The gastrointestinal (GI) prokinetic properties of 5-HT_4_ receptor agonists, developed for GI hypomotility disorders like gastroparesis and constipation (Galligan and Vanner, 2005), are related to interaction with stimulating 5-HT_4_ receptors on cholinergic neurons innervating the GI smooth muscle layer (Gershon and Tack, 2007). The latter 5-HT_4_ receptors, facilitating acetylcholine release and cholinergic contraction, were shown in the GI tract of several species: guinea-pig (Craig and Clarke, 1990; Tonini et al., 1992), rat (Cellek et al., 2008), dog (Prins et al., 2000, 2001), pig (Priem and Lefebvre, 2011; Priem et al., 2012), and man (Prins et al., 2000; Leclere and Lefebvre, 2002; Leclere et al., 2005; Cellek et al., 2006).

5-HT_4_ receptors are G_s_ protein-coupled receptors linked to adenylyl cyclase and the generation of cAMP (Raymond et al., 2001). Phosphodiesterases (PDEs) are the sole family of enzymes degrading the cyclic nucleotides cAMP and cGMP. The intraneuronal transduction pathway of stimulating 5-HT_4_ receptors on cholinergic neurons is regulated by cAMP specific PDE4 in circular muscle of porcine stomach (Priem et al., 2012; Lefebvre et al., 2016) and colon (Priem et al., 2013). Selective PDE4 inhibition by rolipram enhanced the facilitation by 5-HT_4_ receptor stimulation of electrically induced acetylcholine release and cholinergic contraction in circular muscle strips. We recently showed that PDE4 inhibition with rolipram or roflumilast also enhances the facilitating effect of the 5-HT_4_ receptor agonist prucalopride on acetylcholine release in circular muscle strips of human large intestine (Pauwelyn et al., 2018). This suggests the possible usefulness of combining a 5-HT_4_ receptor agonist with a PDE4 inhibitor in man, allowing enhanced GI prokinetic effects of the 5-HT_4_ receptor agonist in low dose. When considering this combination, two possible interferences have to be considered. First, 5-HT_4_ receptors are also present in porcine and human atria (Parker et al., 1995); their stimulation only induces weak chrono- and inotropic effects but they are strictly controlled by PDEs and under PDE inhibition, effects become pronounced (Brattelid et al., 2004; De Maeyer et al., 2006). However, porcine cardiac 5-HT_4_ receptors are under redundant control of PDE3 plus PDE4 (Galindo-Tovar et al., 2009; Weninger et al., 2012) and human cardiac 5-HT_4_ receptors are under sole control of PDE3 (Afzal et al., 2008) so that PDE4 inhibition will not induce cardiac side effects of the 5-HT_4_ receptor agonist. Second, PDEs are also involved in controlling the cyclic nucleotide turnover in particular cAMP at the level of intestinal smooth muscle; PDE inhibition increases the cAMP content leading to inhibition of cholinergic contraction (Barnette et al., 1993; Kaneda et al., 2004) which would counteract cholinergic facilitation at the neuronal level. But in porcine colon, PDE3 is the main regulator of circular smooth muscle activity and PDE4 inhibition hardly influenced cholinergic contractions (Priem et al., 2013). If this would also be the case in man, the combination of a PDE4 inhibitor with a 5-HT_4_ receptor agonist can be considered for GI hypomotility.

Before taking the step to humans, the prokinetic properties of the combination should be tested *in vivo* in an experimental model. The pig is expensive, not easily accessible, and more difficult to handle *in vivo*. Recently it was shown that 5-HT_4_ receptors also facilitate cholinergic nerves innervating circular muscle in fundus, jejunum, and colon of C57Bl/6J mice (Pauwelyn and Lefebvre, 2017). Whether PDE4 is also selectively regulating the intraneuronal signal transduction of these receptors in the murine GI tract and what PDEs are involved in the regulation of the cAMP content in murine GI circular muscle is not known. The aim of this study was therefore to investigate the role of cAMP catalyzing PDEs in murine GI circular muscle activity, and in the signal transduction of the 5-HT_4_ receptors on the cholinergic nerves to the murine GI circular muscle layer. This was done by investigating the influence of selective PDE inhibitors on cholinergic contractions induced with exogenous carbachol or by endogenous acetylcholine released by electrical field stimulation (EFS), and on submaximal facilitation of EFS-induced cholinergic contractions with the 5-HT_4_ receptor agonist prucalopride.

## Materials and Methods

### Animals and Tissue Preparation

All experimental procedures were approved by the Ethical Committee for Animal Experiments from the Faculty of Medicine and Health Sciences at Ghent University. Male C57Bl/6J mice (minimal 7 weeks, body weight 24.2 ± 0.1 g, mean ± SEM of *n* = 245) were purchased from Janvier Labs (Le Genest-Saint-Isle, France) and maintained on normal light-dark cycle with food pellets and water *ad libitum*.

Mice were sacrificed by cervical dislocation; the GI tract was isolated and kept in aerated (95% O_2_ + 5% CO_2_) Krebs solution containing (in mM): NaCl 118.5, KCl 4.8, KH_2_PO_4_ 1.2, MgSO_4_ 1.2, CaCl_2_ 1.9, NaHCO_3_ 25.0, and glucose 10.1. After emptying the stomach from its contents, two full thickness fundus strips were prepared by cutting in the direction of the circular smooth muscle layer. For jejunum an approximately 5 cm long segment of the small bowel, starting 10 cm distally to the pylorus, and for distal colon an approximately 4 cm long segment above the pelvic brim was taken. Jejunum and colon segments were opened along the mesenteric border and pinned with the mucosa side up in Krebs solution. By sharp dissection the mucosa was removed under a microscope and 2 (colon) or 4 (jejunum) smooth muscle strips were cut along the circular axis.

A cotton (fundus) or silk thread (jejunum and colon) was attached to both ends of every strip and they were mounted between two platinum plate electrodes (6–8 mm apart) in a 7 or 15 ml organ bath filled with oxygenated Krebs solution kept at body temperature. EFS was applied with a 4-channel custom-made stimulator. Changes in muscle tone were registered by isometric tension recording using Grass FT03 (Grass Instrument Co., Quincy, MA, United States) or MLT 050/D force transducers (ADInstruments, Oxford, United Kingdom) and recorded on a Powerlab/8sp data recording system (ADInstruments) with Chart v5.5.6 software (ADInstruments). The wet weight of the strips was determined at the end of the experiments.

### Protocols

Strips were set at optimal load of 1 g for fundus, 0.25 g for jejunum, and 0.5 g for colon as previously determined by studying the contractile response to the muscarinic receptor agonist carbachol (Pauwelyn and Lefebvre, 2017) and equilibrated for 30 min in Krebs solution with flushing every 10 min. Contractions were then induced with carbachol (0.1 μM for fundus and jejunum, and 1 μM for colon); within 3 min the contractions reached a plateau. Addition of carbachol was repeated respecting an interval of 20 min after washout of carbachol (with additional flushing after 10 min) until stable responses (defined as a change in amplitude < 25% between consecutive contractions) were obtained. Further submaximal contractions were then induced by carbachol, as described in the Section “Influence of PDE Inhibitors on Carbachol-Induced Submaximal Contractions,” or by EFS, as described in the Section “Influence of PDE Inhibitors on EFS-Induced Submaximal Cholinergic Contractions.”

#### Influence of PDE Inhibitors on Carbachol-Induced Submaximal Contractions

After obtaining stable responses to carbachol, a submaximal concentration of carbachol was added: 0.04 (fundus), 0.05 (jejunum), or 0.3 (colon) μM as based on preliminary concentration-response curves. The responses reached a plateau after 3 min, which corresponded to approximately 50–65% of the contraction amplitude observed with the carbachol concentration used to stabilize the strip. Carbachol was washed-out and after 10 min the organ bath content was flushed a second time. Addition of carbachol was repeated with an interval of 30 min until stable responses to carbachol (defined as a change in amplitude < 25% between consecutive contractions) were obtained. Some strips were excluded from data analysis since the response to carbachol continued to increase with still > 25% from a 4^th^ to 5^th^ addition of carbachol, the maximal number of carbachol additions being kept at 5 (13 out of 69 fundus, 5 out of 71 jejunal, and 4 out of 64 colon strips). A first concentration of PDE inhibitor (0.01 μM) was then added and left in contact with the tissue for 20 min before administering carbachol again. The cycle was then repeated 7 times, increasing the concentration of PDE inhibitor with half-log units to a maximum of 30 μM. The PDE inhibitors tested were IBMX, vinpocetine, EHNA, cilostamide, and rolipram; parallel tissues received the corresponding amount of solvent (ethanol, solvent of IBMX; or DMSO, solvent of vinpocetine, cilostamide, and rolipram) or no PDE inhibitor or solvent (pure time control). The eight experimental conditions were rotated over the organ baths from experiment to experiment.

#### Influence of PDE Inhibitors on EFS-Induced Submaximal Cholinergic Contractions

When stable responses to carbachol were obtained, the Krebs solution was switched to Krebs containing 4 μM guanethidine (noradrenergic neuron blocker) and 300 μM N_ω_-nitro-L-arginine methyl ester hydrochloride (L-NAME; nitric oxide synthase inhibitor) to avoid relaxant influences due to noradrenaline and nitric oxide, respectively. For colon tissue, also 1 μM MRS 2500 (P2Y_1_ receptor antagonist) was added to avoid influences of the relaxant neurotransmitter ATP. These conditions were previously shown to allow induction of pure cholinergic on-contractions by EFS (Pauwelyn and Lefebvre, 2017). After 60 min of equilibration (flushing every 15 min), submaximal cholinergic contractions by EFS were obtained as follows. First, 10 s trains of EFS with a pulse width of 0.5 ms, frequency of 4 (fundus), or 8 (jejunum and colon) Hz at supramaximal voltage (V_max_; 30 V) were repeated at 3 (fundus and colon) or 6 (jejunum) min interval; reproducible contractions were obtained within 10 (fundus and colon) or 5 (jejunum) EFS trains. The voltage was then gradually reduced until the amplitude of the contraction was approximately 50% of that obtained at V_max_ (V_50%_); stable responses to EFS at V_50%_ were obtained within 10 (fundus and colon) or 5 (jejunum) trains of EFS. EFS at V_50%_ was then continued at 5 (fundus and colon) or 10 (jejunum) min interval to register five stable reference contractions.

To study the influence of IBMX, it was administered to 3 parallel strips (1, 3, and 10 μM); 10 (fundus and colon) or 5 (jejunum) trains of EFS were then applied in its presence. The 4^th^ parallel strip did not receive IBMX (pure time control); in a separate series the influence of ethanol (solvent of IBMX) was tested. For jejunum, also 0.1, 0.3, and 1 μM IBMX were tested with the same protocol.

To study the influence of the selective PDE inhibitors vinpocetine, EHNA, cilostamide or rolipram, they were added cumulatively with half-log unit increments (0.01–30 μM), applying 5 (fundus and colon) or 3 (jejunum) trains of EFS at each concentration. The corresponding amount of DMSO (solvent of vinpocetine, cilostamide, and rolipram) was also investigated as well as a pure time control. The six experimental conditions were rotated over the organ baths from experiment to experiment.

#### Influence of PDE Inhibitors on the Effect of Prucalopride on EFS-Induced Submaximal Cholinergic Contractions

These experiments started as described in the Section “Influence of PDE Inhibitors on EFS-Induced Submaximal Cholinergic Contractions” till five stable contractions by EFS at V_50%_ were obtained. The influence of the PDE inhibitors was then tested on the facilitating effect of a submaximal concentration of prucalopride (0.003 μM; Pauwelyn and Lefebvre, 2017). The concentrations of the PDE inhibitors studied were selected from their effect on carbachol- and EFS-induced contractions (see the section “Results”); a single concentration of PDE inhibitor was tested per strip. After adding the PDE inhibitor, 10 (fundus and colon) or 5 (jejunum) trains of EFS were applied; prucalopride was then added and a further series of 10 or 5 trains were applied. Parallel strips received either prucalopride alone, or no active compounds (pure time control). The experimental conditions were rotated over the organ baths from experiment to experiment.

### Data and Statistical Analysis

The contraction force induced by carbachol or EFS was expressed as gram.second per milligram wet weight of the tissue (g.s/mg wet weight). This was calculated by determining the area under the curve (AUC) during the contraction (3 min for carbachol- and 10 s for EFS-induced contractions) reduced with the AUC during a corresponding time period just before adding carbachol or applying EFS.

Carbachol-induced contractions in the presence of substances were expressed as percentage of the last contraction just before addition (100% reference). EFS-induced contractions at V_50%_ in the presence of substances were expressed as percentage of the mean of the five stable contractions just before addition (100% reference). For experiments, testing the influence of PDE inhibitors on the effect of prucalopride, the contractions after addition of prucalopride were also expressed as percentage of the mean of the five contractions in the presence of the PDE inhibitor just before adding prucalopride.

Strips where the AUC of minimally one of the five reference contractions by EFS used to calculate the mean 100% reference was outside the range of 75–125%, were excluded from data analysis. As an example of the exclusion rate, in the series studying IBMX on EFS-induced submaximal contractions, 4 out of 24 fundus and 7 out of 48 jejunum strips were excluded; none of the 16 colon strips had to be excluded. Experiments testing the influence of PDE inhibitors on the effect of prucalopride were excluded from data analysis when the response in the presence of 0.003 μM prucalopride alone was lower than 115% for fundus, 107% for jejunum, and 116% for colon (as these are the minimal effects for this concentration of prucalopride, observed in our previous study in the murine GI tract; Pauwelyn and Lefebvre, 2017).

Data are expressed as means ± SEM; *n* refers to tissues obtained from different animals. Statistical analysis was performed by use of GraphPad Prism version 5.03 (GraphPad Software, San Diego, CA, United States). In experiments with cumulative administration of PDE inhibitor, carbachol- and EFS-induced contractions were assessed with a repeated measures ANOVA followed by a Bonferroni corrected *t*-test for comparison to the reference contraction in the same strips or with an unpaired *t*-test for comparison with the corresponding control strips. For experiments with single administration of PDE inhibitor, EFS-induced contractions induced by the last train of EFS in parallel groups were compared by a one-way ANOVA followed by a Bonferroni corrected *t*-test. A *P*-value less than 0.05 was considered statistically significant.

### Drugs

Carbamoylcholine chloride, guanethidine sulfate, 3-isobutyl-1-methylxanthine (IBMX) and N_ω_-nitro-L-arginine methyl ester hydrochloride (L-NAME) were obtained from Sigma–Aldrich (St. Louis, MO, United States); cilostamide, erythro-9-(2-hydroxy-3-nonyl)adenine (EHNA) hydrochloride, MRS 2500 tetraammonium salt [(1R^∗^,2S^∗^)-4-[2-Iodo-6-(methylamino)-9H -purin -9-yl]-2-(phosphonooxy)bicyclo[3.1.0]hexane-1-me-thanol dihydrogen phosphate ester tetraammonium salt], rolipram and vinpocetine from Tocris Bioscience (Bristol, United Kingdom), and prucalopride succinate from Selleck Chemicals (Houston, TX, United States). Drugs were dissolved and diluted in distilled water, except for IBMX which was dissolved in ethanol, yielding a maximal ethanol concentration of 0.15% in the organ bath, and vinpocetine, cilostamide, and rolipram which were dissolved in dimethyl sulfoxide (DMSO), yielding a maximal DMSO concentration of 0.3% in the organ bath.

## Results

### Influence of PDE Inhibitors on Carbachol-Induced Submaximal Contractions (**Figures 1–3**)

**FIGURE 1:**
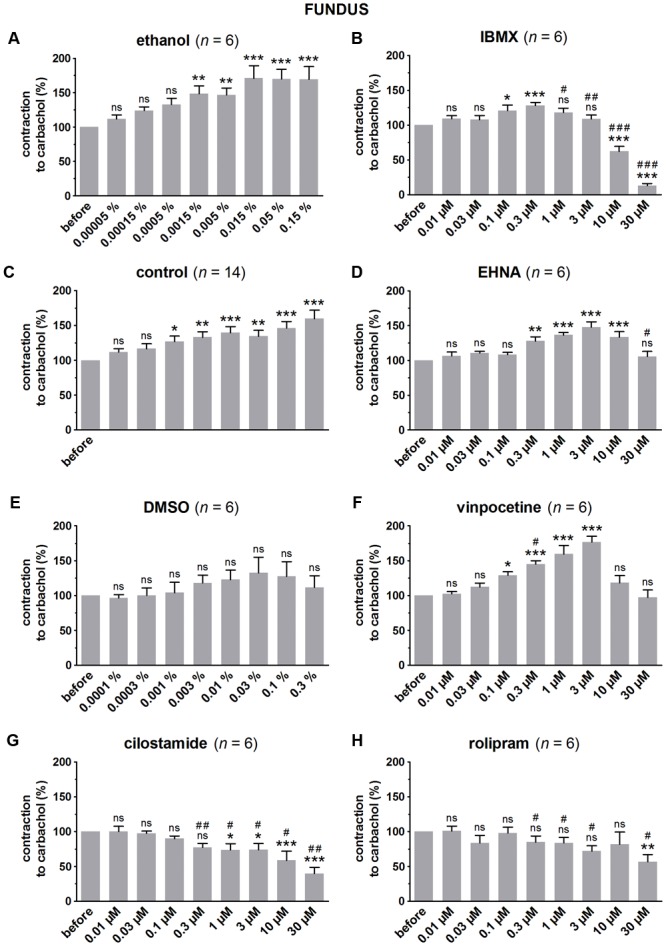
Influence of increasing concentrations (0.01–30 μM) of the PDE inhibitors IBMX **(B)**, EHNA **(D)**, vinpocetine **(F)**, cilostamide **(G)** and rolipram **(H)**, and the solvents ethanol **(A)** and DMSO **(E)** on submaximal carbachol-induced contractions (0.04 μM) in murine fundus circular smooth muscle strips. Parallel time controls not receiving PDE inhibitor or solvent are shown in **(C)**. Contractions in the presence of increasing concentrations of the PDE inhibitor are expressed as percentage of the reference contraction before addition of the PDE inhibitor. Means ± SEM; ns not significant, ^∗^ *P* < 0.05, ^∗∗^ *P* < 0.01, ^∗∗∗^ *P* < 0.001 versus reference before (repeated measures ANOVA with Bonferroni corrected *t*-test) and ^#^ *P* < 0.05, ^##^ *P* < 0.01, ^###^ *P* < 0.001 versus corresponding control (unpaired *t*-test).

**FIGURE 2:**
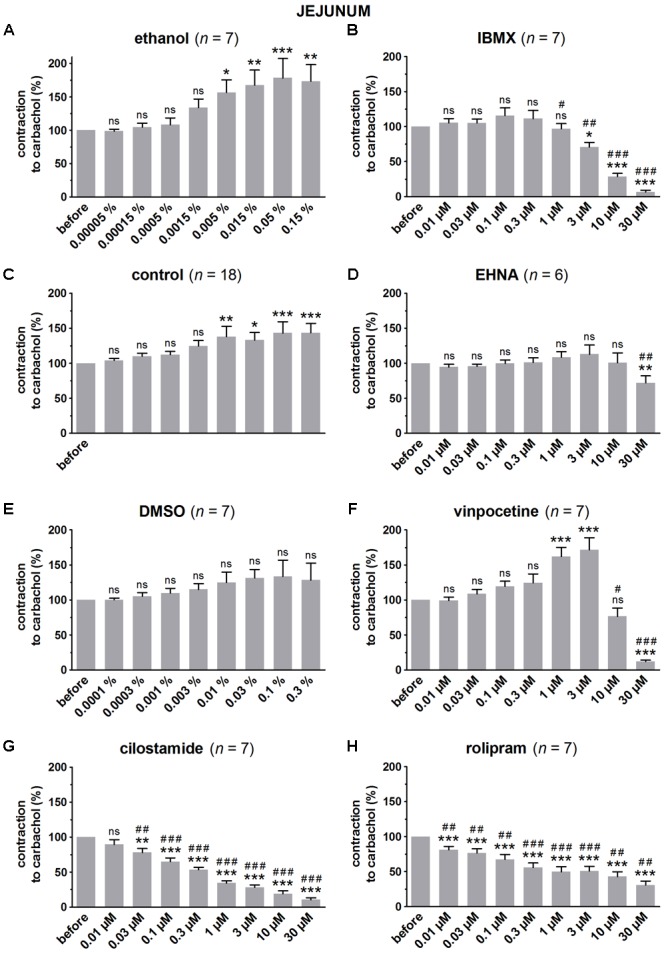
Influence of increasing concentrations (0.01–30 μM) of the PDE inhibitors IBMX **(B)**, EHNA **(D)**, vinpocetine **(F)**, cilostamide **(G)** and rolipram **(H)**, and the solvents ethanol **(A)** and DMSO **(E)** on submaximal carbachol-induced contractions (0.05 μM) in murine jejunum circular smooth muscle strips. Parallel time controls not receiving PDE inhibitor or solvent are shown in **(C)**. Contractions in the presence of increasing concentrations of the PDE inhibitor are expressed as percentage of the reference contraction before addition of the PDE inhibitor. Means ± SEM; ns not significant, ^∗^ *P* < 0.05, ^∗∗^ *P* < 0.01, ^∗∗∗^ *P* < 0.001 versus reference before (repeated measures ANOVA with Bonferroni corrected *t*-test) and ^#^ *P* < 0.05, ^##^ *P* < 0.01, ^###^ *P* < 0.001 versus corresponding control (unpaired *t*-test).

**FIGURE 3:**
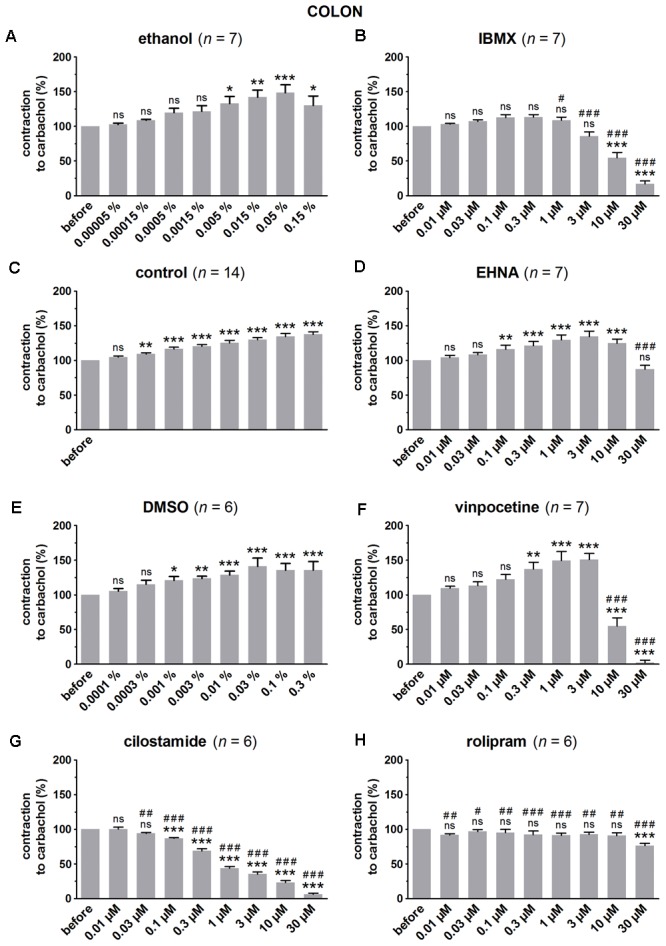
Influence of increasing concentrations (0.01–30 μM) of the PDE inhibitors IBMX **(B)**, EHNA **(D)**, vinpocetine **(F)**, cilostamide **(G)** and rolipram **(H)**, and the solvents ethanol **(A)** and DMSO **(E)** on submaximal carbachol-induced contractions (0.3 μM) in murine colon circular smooth muscle strips. Parallel time controls not receiving PDE inhibitor or solvent are shown in **(C)**. Contractions in the presence of increasing concentrations of the PDE inhibitor are expressed as percentage of the reference contraction before addition of the PDE inhibitor. Means ± SEM; ns not significant, ^∗^ *P* < 0.05, ^∗∗^ *P* < 0.01, ^∗∗∗^ *P* < 0.001 versus reference before (repeated measures ANOVA with Bonferroni corrected *t*-test) and ^#^ *P* < 0.05, ^##^ *P* < 0.01, ^###^ *P* < 0.001 versus corresponding control (unpaired *t*-test).

Since the non-selective PDE inhibitor IBMX is dissolved in ethanol, the control strips received the corresponding amount of ethanol (**Figures 1A, 2A, 3A**). In the presence of an increasing concentration of ethanol, the carbachol-induced contractions steadily increased. This is not due to ethanol because this trend was also present in pure time control strips not receiving solvent or PDE inhibitor (**Figures 1C, 2C, 3C**). When comparing the effect of IBMX with the corresponding control strips receiving ethanol, IBMX concentration-dependently decreased the carbachol-induced contractions from 1 μM onwards in the strips from the three GI regions (**Figures 1B, 2B, 3B**).

The selective PDE2 inhibitor EHNA is dissolved in H_2_O and can thus be compared with the pure time controls. Only the highest concentration of EHNA induced a significant decrease of carbachol-induced contraction in fundus (**Figure 1D**), jejunum (**Figure 2D**), and colon (**Figure 3D**). Vinpocetine, cilostamide and rolipram, respectively, selective PDE1, PDE3, and PDE4 inhibitors, were dissolved in DMSO. Compared with the control strips receiving the corresponding amount of DMSO (**Figures 1E, 2E, 3E**), a clear-cut concentration-dependent decrease of carbachol-induced contraction was obtained with cilostamide starting at 0.3 μM in the fundus (**Figure 1G**) and at 0.03 μM in jejunum and colon (**Figures 2G, 3G**). Also rolipram concentration-dependently decreased the carbachol-induced contractions over a broad concentration span in strips from the three GI regions (**Figures 1H, 2H, 3H**), but the decrease was less pronounced compared to cilostamide in particular in the colon. Vinpocetine significantly decreased the contractions in jejunum (**Figure 2F**) and colon (**Figure 3F**) in the two highest concentrations tested (10–30 μM), but not in the fundus although a similar trend is visible (**Figure 1F**).

### Influence of PDE Inhibitors on EFS-Induced Submaximal Contractions (**Figures 4–7** and **Table 1A**)

**FIGURE 4:**
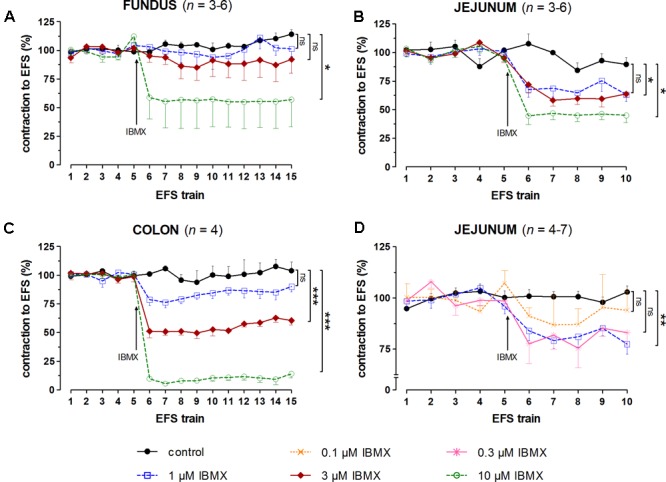
Influence of 1, 3, and 10 μM **(A–C)** and 0.1, 0.3, and 1 μM **(D)** IBMX on submaximal electrically induced cholinergic contractions at V_50%_ (10 s trains at 4 [fundus] or 8 [jejunum and colon] Hz, 0.5 ms, interval of 5 [fundus and colon] or 10 [jejunum] min) in murine fundus **(A)**, jejunum **(B,D)**, and colon **(C)** circular smooth muscle strips. The number of the consecutive stimulation trains before and after adding IBMX is given on the y-axis. Contractions are expressed as percentage of the mean of the five contractions before adding IBMX (trains 1–5). Experiments were performed in the continuous presence of 4 μM guanethidine, 300 μM L-NAME, and for colon also 1 μM MRS 2500. Means ± SEM; ns not significant, ^∗^ *P* < 0.05, ^∗∗^ *P* < 0.01, ^∗∗∗^ *P* < 0.001 versus control (one-way ANOVA with Bonferroni corrected *t*-test for the 3 concentrations of IBMX versus control).

**FIGURE 5:**
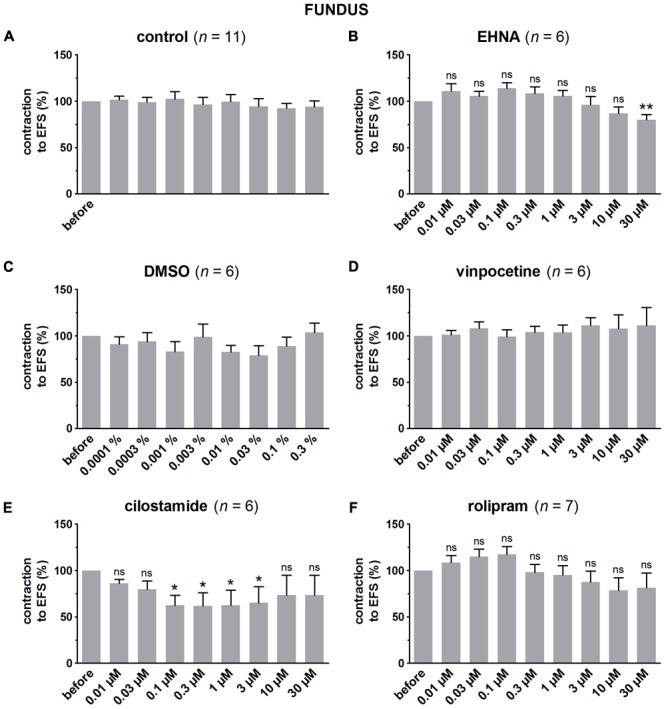
Influence of increasing concentrations (0.01–30 μM) of the PDE inhibitors EHNA **(B)**, vinpocetine **(D)**, cilostamide **(E)** and rolipram **(F)**, and the solvent DMSO **(C)** on submaximal electrically induced cholinergic contractions at V_50%_ (10 s trains at 4 Hz, 0.5 ms, interval of 5 min) in murine fundus circular smooth muscle strips. Parallel time controls not receiving PDE inhibitor or solvent are shown in **(A)**. Five contractions were induced in the presence of each concentration of the PDE inhibitor and every 5^th^ contraction is shown, expressed as percentage of the mean of the five contractions before addition of the PDE inhibitor. Experiments were performed in the continuous presence of 4 μM guanethidine and 300 μM L-NAME. Means ± SEM; ns not significant, ^∗^ *P* < 0.05, ^∗∗^*P* < 0.01 versus reference before (repeated measures ANOVA with Bonferroni corrected *t*-test). For control, DMSO, and vinpocetine, the repeated measures ANOVA was not significant.

**FIGURE 6:**
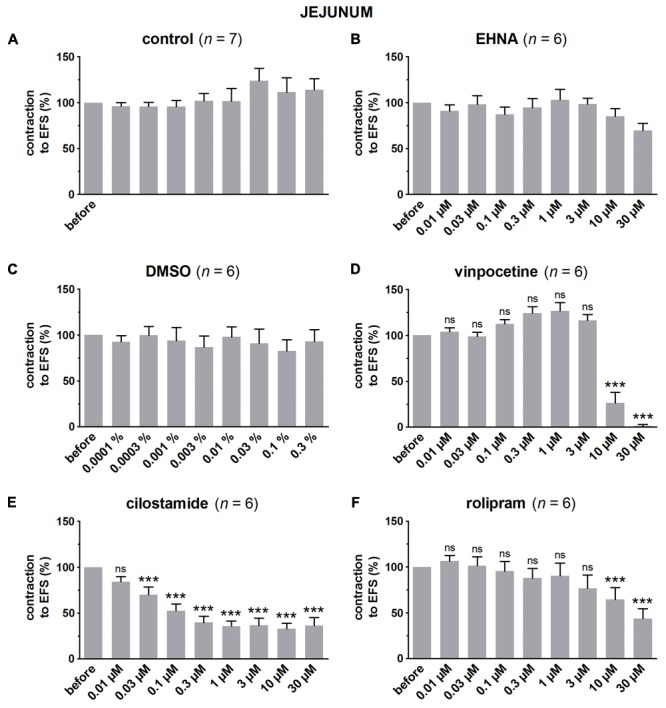
Influence of increasing concentrations (0.01–30 μM) of the PDE inhibitors EHNA **(B)**, vinpocetine **(D)**, cilostamide **(E)** and rolipram **(F)**, and the solvent DMSO **(C)** on submaximal electrically induced cholinergic contractions at V_50%_ (10 s trains at 8 Hz, 0.5 ms, interval of 10 min) in murine jejunum circular smooth muscle strips. Parallel time controls not receiving PDE inhibitor or solvent are shown in **(A)**. Three contractions were induced in the presence of each concentration of the PDE inhibitor and every 3^rd^ contraction is shown, expressed as percentage of the mean of the five contractions before addition of the PDE inhibitor. Experiments were performed in the continuous presence of 4 μM guanethidine and 300 μM L-NAME. Means ± SEM; ns not significant, ^∗∗∗^ *P* < 0.001 versus reference before (repeated measures ANOVA with Bonferroni corrected *t*-test). For control, EHNA, and DMSO, the repeated measures ANOVA was not significant.

**FIGURE 7:**
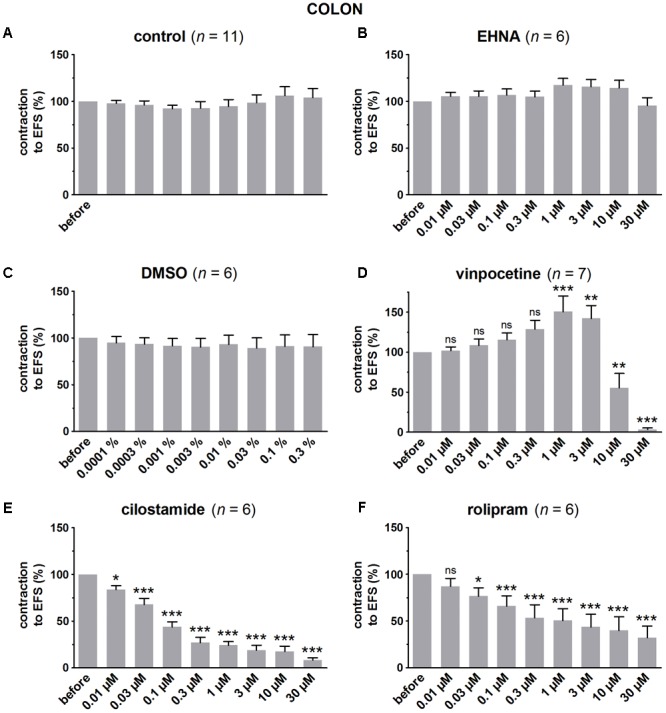
Influence of increasing concentrations (0.01–30 μM) of the PDE inhibitors EHNA **(B)**, vinpocetine **(D)**, cilostamide **(E)** and rolipram **(F)**, and the solvent DMSO **(C)** on submaximal electrically induced cholinergic contractions at V_50%_ (10 s trains at 8 Hz, 0.5 ms, interval of 5 min) in murine colon circular smooth muscle strips. Parallel time controls not receiving PDE inhibitor or solvent are shown in **(A)**. Five contractions were induced in the presence of each concentration of the PDE inhibitor and every 5^th^ contraction is shown, expressed as percentage of the mean of the five contractions before addition of the PDE inhibitor. Experiments were performed in the continuous presence of 4 μM guanethidine, 300 μM L-NAME, and 1 μM MRS 2500. Means ± SEM; ns not significant, ^∗^ *P* < 0.05, ^∗∗^ *P* < 0.01, ^∗∗∗^ *P* < 0.001 versus reference before (repeated measures ANOVA with Bonferroni corrected *t*-test). For control, EHNA, and DMSO, the repeated measures ANOVA was not significant.

**Table 1:** Influence of IBMX on EFS-induced submaximal cholinergic contractions **(A)** and on their facilitation by prucalopride **(B)** in murine fundus, jejunum, and colon. Mean ± SEM.

	fundus	jejunum	colon
A – IBMX on EFS-induced contractions (%)			
control	114 ± 4 (*n* = 6)	90 ± 6 (*n* = 3)	104 ± 8 (*n* = 4)
1 μM IBMX	101 ± 6 ^ns^ (*n* = 5)	63 ± 6 ^ns^ (*n* = 4)	90 ± 4 ^ns^ (*n* = 4)
3 μM IBMX	92 ± 12 ^ns^ (*n* = 6)	64 ± 2 ^∗^ (*n* = 5)	61 ± 4 ^∗∗∗^ (*n* = 4)
10 μM IBMX	57 ± 24 ^∗^ (*n* = 3)	45 ± 6 ^∗^ (*n* = 6)	14 ± 4 ^∗∗∗^ (*n* = 4)
**B – IBMX on effect of prucalopride on EFS-induced contractions (%)**			
control	102 ± 8 (*n* = 8)	95 ± 5 (*n* = 8)	111 ± 2 (*n* = 7)
0.003 μM prucalopride	138 ± 6 ^ns^ (*n* = 9)	128 ± 5 ^∗∗∗^ (*n* = 9)	141 ± 11 ^∗^ (*n* = 7)
0.1 μM IBMX + 0.003 μM prucalopride	ND	127 ± 4 ^ns^ (*n* = 7)	ND
0.3 μM IBMX + 0.003 μM prucalopride	ND	120 ± 4 ^ns^ (*n* = 7)	129 ± 7 ^ns^ (*n* = 7)
1 μM IBMX + 0.003 μM prucalopride	150 ± 14 ^ns^ (*n* = 7)	ND	127 ± 7 ^ns^ (*n* = 6)
3 μM IBMX + 0.003 μM prucalopride	163 ± 14 ^ns^ (*n* = 9)	ND	ND

***A** – Contractile response to the 5^*th*^ (jejunum) or 10^*th*^ (fundus and colon) train of EFS in the presence of IBMX as percentage of the mean of the five contractions before adding IBMX. ^*ns*^ not significant, ^∗^*P* < 0.05, ^∗∗∗^*P* < 0.001 versus control (one-way ANOVA with Bonferroni corrected *t*-test for the 3 concentrations of IBMX versus control). **B** – Contractile response to the 5^*th*^ (jejunum) or 10^*th*^ (fundus and colon) train of EFS in the presence of prucalopride as percentage of the mean of the five contractions in the presence of IBMX just before adding prucalopride. ND: Not determined. ^*ns*^ not significant, ^∗^*P* < 0.05, ^∗∗∗^*P* < 0.001 (one-way ANOVA with Bonferroni corrected *t*-test for the three comparisons i.e., prucalopride versus control, and both concentrations of IBMX versus prucalopride).*

In control strips from the three GI regions, a fairly stable response to EFS was obtained (**Figure 4**). IBMX, 1, 3, and 10 μM, concentration-dependently decreased the EFS-induced submaximal cholinergic contractions (**Figures 4A,B,C** and **Table 1A**) in the three regions. As 1 μM IBMX, the lowest concentration tested, had a more pronounced effect on EFS-induced submaximal cholinergic contractions in jejunum than in fundus and colon, 10-fold lower concentrations were also investigated (**Figure 4D**). These induced concentration-dependent decreases from 103 ± 3% for control strips to 94 ± 9% with 0.1 μM, 83 ± 6% with 0.3 μM, and 77 ± 5% (*P* < 0.01) with 1 μM IBMX. In a separate series, the solvent of IBMX (ethanol) did not show any influence (results not shown).

In pure time controls (**Figures 5A, 6A, 7A**), but also in strips receiving increasing amounts of DMSO (**Figures 5C, 6C, 7C**), a fairly stable response to EFS was induced for the three GI regions. Therefore, the statistical analysis was restricted to the comparison of the last EFS-induced contraction in the presence of each concentration of a given PDE inhibitor with the mean of the five contractions before addition of the PDE inhibitor. In the studied concentration range (0.01–30 μM) EHNA (**Figures 5B, 6B, 7B**) only mildly reduced the EFS-induced contractions at 30 μM in the fundus and was without effect in jejunum and colon, while vinpocetine (**Figures 5D, 6D, 7D**) had no influence in the fundus but reduced the EFS-induced contractions at the two highest concentrations tested in jejunum and colon. Cilostamide (**Figures 5E, 6E, 7E**) decreased the contractions over a broad concentration range in the strips from the three GI regions but no clear concentration-dependency was present in the fundus. Rolipram did not significantly influence the contractions in the fundus (**Figure 5F**), decreased them in the jejunum (**Figure 6F**) at the two highest concentrations studied and concentration-dependently decreased them from 0.03 μM onwards in the colon (**Figure 7F**). The reduction of the contractions with rolipram in the colon was less pronounced than obtained with cilostamide.

### Influence of PDE Inhibitors on the Effect of Prucalopride on EFS-Induced Submaximal Cholinergic Contractions (**Figures 8–10, Table 1B**, and Supplementary Figures 1–4)

**FIGURE 8:**
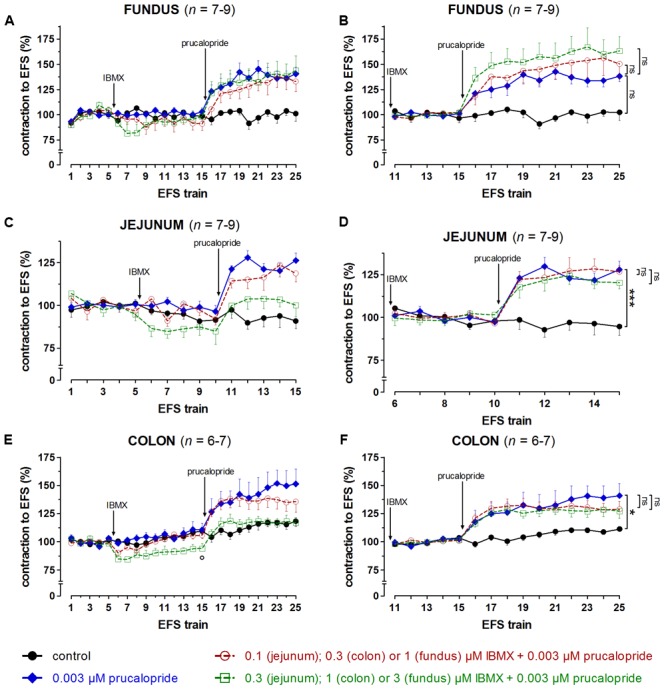
Influence of IBMX in a concentration of 1 and 3 μM (fundus), 0.1 and 0.3 μM (jejunum), and 0.3 and 1 μM (colon) on the facilitating effect of 0.003 μM prucalopride on submaximal electrically induced cholinergic contractions at V_50%_ (10 s trains at 4 [fundus] or 8 [jejunum and colon] Hz, 0.5 ms, interval of 5 [fundus and colon] or 10 [jejunum] min) in murine fundus **(A,B)**, jejunum **(C,D)**, and colon **(E,F)** circular smooth muscle strips. The number of the consecutive stimulation trains is given on the y-axis. Contractions are expressed as percentage of the mean of the five contractions before adding IBMX (trains 1–5; **A,C,E**) or of the five contractions in the presence of IBMX just before adding prucalopride (trains 11–15 for fundus and colon; trains 6–10 for jejunum; **B,D,F**). Experiments were performed in the continuous presence of 4 μM guanethidine, 300 μM L-NAME, and for colon also 1 μM MRS 2500. Means ± SEM. Left panels, last EFS-induced contraction in the presence of PDE inhibitor before adding prucalopride: °*P* < 0.05 versus control (unpaired *t*-test). Right panels, last EFS-induced contraction: ns not significant, ^∗^ *P* < 0.05, ^∗∗∗^ *P* < 0.001 (one-way ANOVA with Bonferroni corrected *t*-test for three comparisons i.e., prucalopride versus control and both concentrations of IBMX versus prucalopride).

**FIGURE 9:**
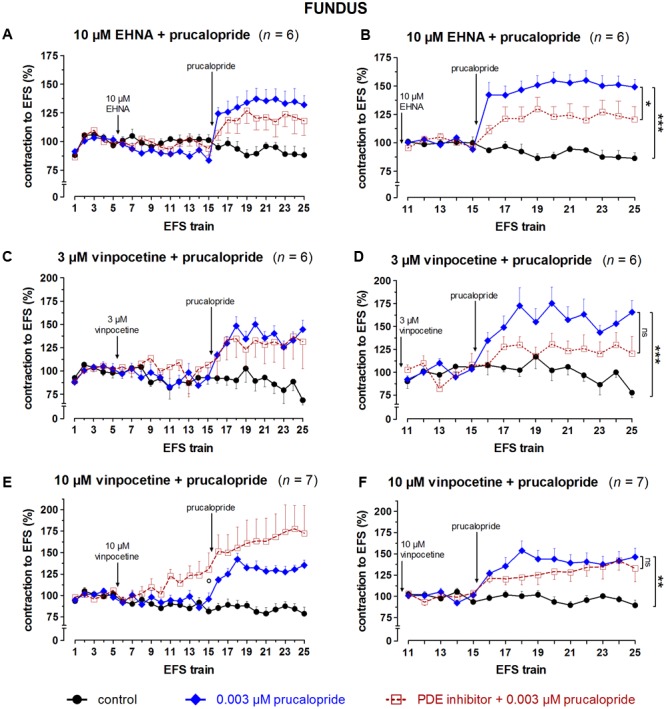
Influence of 10 μM EHNA **(A,B)**, 3 **(C,D)** and 10 **(E,F)** μM vinpocetine on the facilitating effect of 0.003 μM prucalopride on submaximal electrically induced cholinergic contractions at V_50%_ (10 s trains at 4 Hz, 0.5 ms, interval of 5 min) in murine fundus circular smooth muscle strips. The number of the consecutive stimulation trains is given on the y-axis. Contractions are expressed as percentage of the mean of the five contractions before addition of the PDE inhibitor (trains 1–5; **A,C,E**) or of the five contractions in the presence of the PDE inhibitor just before adding prucalopride (trains 11–15; **B,D,F**). Experiments were performed in the continuous presence of 4 μM guanethidine and 300 μM L-NAME. Means ± SEM. Left panels, last EFS-induced contraction before adding prucalopride: ° *P* < 0.05 versus control (one-way ANOVA with Bonferroni corrected *t*-test for two comparisons i.e., PDE inhibitor versus control and versus prucalopride). Right panels, last EFS-induced contraction: ns not significant, ^∗^ *P* < 0.05, ^∗∗^ *P* < 0.01, ^∗∗∗^ *P* < 0.001 (one-way ANOVA with Bonferroni corrected *t*-test for two comparisons i.e., prucalopride versus control and versus prucalopride in the presence of the PDE inhibitor).

**FIGURE 10:**
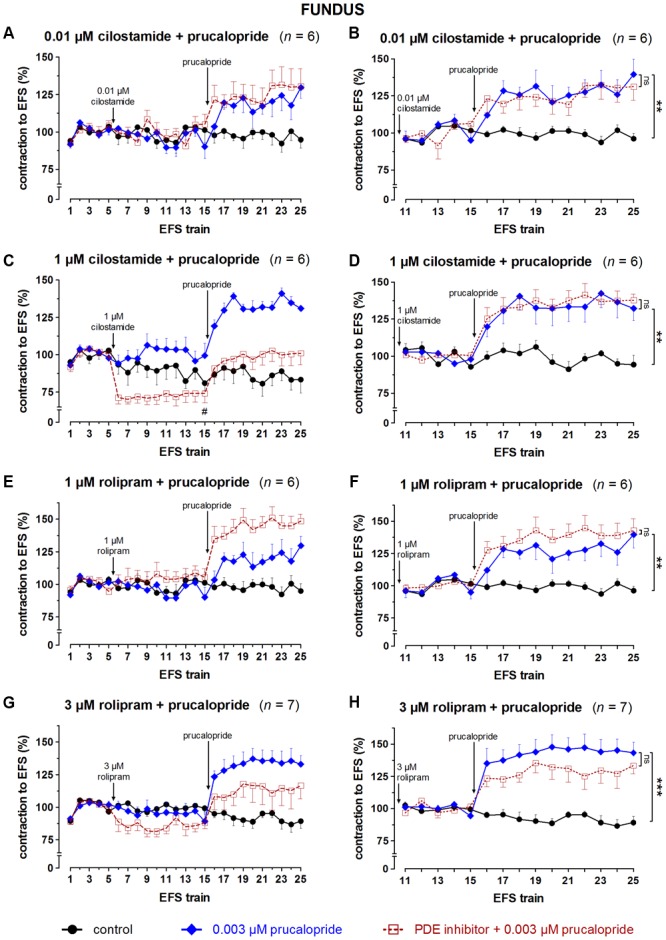
Influence of 0.01 **(A,B)** and 1 **(C,D)** μM cilostamide, 1 **(E,F)** and 3 **(G,H)** μM rolipram on the facilitating effect of 0.003 μM prucalopride on submaximal electrically induced cholinergic contractions at V_50%_ (10 s trains at 4 Hz, 0.5 ms, interval of 5 min) in murine fundus circular smooth muscle strips. The number of the consecutive stimulation trains is given on the y-axis. Contractions are expressed as percentage of the mean of the five contractions before addition of the PDE inhibitor (trains 1–5; **A,C,E,G**) or of the five contractions in the presence of the PDE inhibitor just before adding prucalopride (trains 11–15; **B,D,F,H**). Experiments were performed in the continuous presence of 4 μM guanethidine and 300 μM L-NAME. Means ± SEM. Left panels, last EFS-induced contraction before adding prucalopride: ^#^ *P* < 0.05 versus prucalopride (one-way ANOVA with Bonferroni corrected *t*-test for two comparisons i.e., PDE inhibitor versus control and versus prucalopride). Right panels, last EFS-induced contraction: ns not significant, ^∗∗^ *P* < 0.01, ^∗∗∗^ *P* < 0.001 (one-way ANOVA with Bonferroni corrected *t*-test for two comparisons i.e., prucalopride versus control and versus prucalopride in the presence of the PDE inhibitor).

Taking in account the decreasing effect of IBMX as such on EFS-induced submaximal cholinergic contractions (see the section “Influence of PDE Inhibitors on EFS-Induced Submaximal Contractions (**Figures 4–7** and **Table 1A**)”; **Figure 4** and **Table 1A**), two concentrations of IBMX, inducing less than 20% decrease, were selected to test versus 0.003 μM prucalopride: 1 and 3 μM in fundus, 0.1 and 0.3 μM in jejunum, and 0.3 and 1 μM in colon. Also in this series, the decrease of the EFS-induced contractions by these concentrations of IBMX was lower than 20% (**Figures 8A,C,E**) and only reached significance for 1 μM in the colon (*P* < 0.05 versus controls). Still in view of the effect of IBMX as such, the responses in the presence of prucalopride were also expressed as percentage of the mean of the five contractions just before adding prucalopride (**Figures 8B,D,F** and **Table 1B**). This showed that 0.003 μM prucalopride *per se* facilitated the electrically induced cholinergic contractions, the effect not reaching significance in the fundus, but the presence of IBMX did not significantly enhance the effect of prucalopride although a trend was present in the fundus.

To select the concentrations of the selective PDE inhibitors vinpocetine (PDE1), EHNA (PDE2), cilostamide (PDE3), and rolipram (PDE4) for testing versus prucalopride three elements were taken in account: (1) the effect of the PDE inhibitor as such on the EFS-induced submaximal cholinergic contractions as reported in the Section “Influence of PDE Inhibitors on EFS-Induced Submaximal Contractions (**Figures 4–7** and **Table 1A**)”; (2) the reported IC_50_ values (**Table 2**), (3) a synergistic effect with prucalopride observed with the inhibitor in similar experiments in porcine GI tissue (Priem et al., 2012, 2013). In principle, for a first test the highest concentration in the range tested (0.01 – 30 μM), not influencing EFS-induced contractions in the three GI regions [see the section “Influence of PDE Inhibitors on EFS-Induced Submaximal Contractions (**Figures 4–7** and **Table 1A**)”] was studied for a given PDE inhibitor. EHNA at 30 μM significantly reduced EFS-induced submaximal cholinergic contractions in the fundus, so a concentration of 10 μM was selected to test versus prucalopride; this concentration is 10-fold higher than to equal to reported IC_50_ values at PDE2. Vinpocetine was studied versus prucalopride at 3 μM, as this did not decrease EFS-induced contractions in the three tissue types. As this concentration is clearly below reported IC_50_ values at PDE1, also 10 μM vinpocetine was studied although it clearly reduced EFS-induced contractions in jejunum and colon. The lowest concentration of cilostamide tested versus EFS-induced contractions (0.01 μM) still had some decreasing influence in the colon and was first tested. As this concentration is 3–13-fold lower than reported IC_50_ values at PDE3, cilostamide was also studied versus prucalopride at 1 μM. For rolipram, 1 and 3 μM were tested versus prucalopride as in porcine GI tissue, these concentrations were able to increase the prucalopride-induced facilitation of EFS-induced contractions (Priem et al., 2012, 2013). 1 μM corresponds to the highest IC_50_ value at PDE4 reported in the literature, although a 20-fold lower value (Bolger et al., 1997) and even an exceptionally 1000-fold lower value (Wang et al., 1997) were also reported (**Table 2**). Both 1 and 3 μM were without effect on EFS-induced submaximal cholinergic contractions in the fundus and jejunum but decreased them in the colon.

**Table 2:** Overview of reported IC_50_ values for the PDE inhibitors studied (in bold for PDE subtype, wherefore the inhibitor is considered selective).

PDE inhibitor	IC_50_ (μM)					Reference (see below)
	PDE1	PDE2	PDE3	PDE4	No subtype identified	
IBMX	12	20	2	5		1^#^
	A: 0.8 C: 23	27	11	21		2^#^
	7.1	18.6	7.1	11.4		3^#^
vinpocetine	**23.2**	ND	ND	ND		4^∗^
	**32.0**	69.2	>100	41.3		3^#^
EHNA	ND	**9.2**	ND	ND		4^∗^
	>100	**0.8 (human) 2 (pig)**	>100	>100		5^#^
	>50	**3.5**	>50	>50		6^#^
	>400	**4.3**	>400	310		7^#^
cilostamide	>300	12.5	**A: 0.027 B: 0.05**	88.8		4^∗^
	221	48	**0.13**	99		7^#^
					0.063	8°
rolipram	ND	ND	ND	**0.45**		4^∗^
	ND	ND	ND	**D1: 0.05 D2: 0.05 D3: 0.14/0.32 D4: 0.06/0.05 D5: 0.08/0.59**		9^∗^
	ND	0.5	0.18	**0.4**		10^∗^
	ND	ND	ND	**A: 0.0011 B: 0.0009 C: 0.3246 D: 0.0611**		11^∗^
	A: 50 C:>100	>100	>100	**0.6**		2^#^
	>100	>100	>100	**0.72**		3^#^
	ND	ND	ND	**0.065**		6^#^
					1	12°

*ND: Not determined. IC_*50*_ values obtained in paradigms: ^∗^ with specific PDE expression; ^#^ where PDE subtypes were purified and/or identified on the basis of their regulatory and kinetic properties; ° where no subtype identification was done, as specified below. 1^#^ – Coste and Grondin, 1995: cAMP (PDE1, 3, and 4) or cGMP (PDE2) hydrolysis by purified PDE1 and 3 from bovine aorta and human recombinant PDE2 and 4. 2^#^ – Torphy and Cieslinski, 1990: cAMP (PDE1) and cGMP (PDE2, 3, and 4) hydrolysis by chromatography separated PDE isozymes in canine trachealis. 3^#^ – Saeki and Saito, 1993: cAMP or cGMP hydrolysis by PDE1 to 5 isozymes, separated by chromatography and identified on the basis of their regulatory and kinetic properties (type 1 preferentially hydrolyzed cGMP and was stimulated by Ca^*2*+^-calmodulin; type 2 was stimulated by cGMP and hydrolyzed cAMP and cGMP; type 3 was inhibited by cGMP and preferentially hydrolyzed cAMP; type 4 was cAMP-specific; type 5 was cGMP specific and its activity did not depend on calmodulin), from the supernatant of pig aortic smooth muscle homogenates. 4^∗^ – Sudo et al., 2000: cAMP (PDE2, 3, and 4) or cGMP (PDE1) hydrolysis by recombinant PDE isozymes expressed in insect (Sf9) cells using baculovirus expression system; cDNAs from bovine PDE1B1 and PDE2A1, human PDE3A1, and rat PDE3B1 and PDE4B2. 5^#^ – Podzuweit et al., 1995: cAMP (PDE3 and 4) or cGMP (PDE1 and 2) hydrolysis by PDE1 to 4 isozymes, separated by chromatography and identified on the basis of their regulatory and kinetic properties (PDE1 was stimulated by Ca^*2*+^-calmodulin and PDE2 by cGMP; PDE3 had a high affinity for cAMP and was inhibited by cGMP; PDE4 was cAMP specific), from human and porcine myocardium. 6^#^ – Michie et al., 1996: cAMP hydrolysis by murine thymocyte PDE1, 2, 3, and 4, separated by chromatography. 7^#^ – O’Grady et al., 2002: cAMP hydrolysis by PDE1, 2, 3, and 4 purified from T_*84*_ human colonic epithelial cell line or bovine cardiac muscle; the IC_*50*_ value of cilostamide for PDE3 was tested on Cl secretion in T_*84*_ cells. 8° – Zacher and Carey, 1999: cAMP hydrolysis by phosphodiesterases in swine adipocyte microsomal membrane fraction. 9^∗^ – Bolger et al., 1997: cAMP hydrolysis by monkey COS-7 cells expressing cDNA of human PDE4D isoenzymes. 10^∗^ – Bolger et al., 1993: cAMP hydrolysis by yeast (*Saccharomyces cerevisiae*) extracts expressing cDNA clones for human PDE2 through 4. 11^∗^ – Wang et al., 1997: cAMP hydrolysis by purified human PDE4A, B, C, and D expressed in insect (Sf9) cells using baculovirus expression system. 12° – Schwabe et al., 1976: cAMP hydrolysis in rat brain slices.*

The results obtained with the selective PDE inhibitors in fundus strips are given in **Figures 9, 10**. In the left panels, responses are expressed as percentage of the mean of the five contractions before adding the PDE inhibitor, allowing to judge the effect of the PDE inhibitor on EFS-induced contractions. In the right panels, responses are expressed as percentage of the mean of the five contractions just before adding prucalopride in the presence of PDE inhibitor; values given below for the facilitating effect of prucalopride refer to this way of expression. As judged from the 10^th^ EFS-induced contraction in its presence, EHNA at 10 μM had no influence on EFS-induced submaximal cholinergic contractions when compared to the two parallel strip groups i.e., the pure time controls and the strips going to receive prucalopride in the absence of the PDE inhibitor (**Figure 9A**). Prucalopride, 0.003 μM, significantly enhanced the contractions to 149 ± 6% (*P* < 0.001 versus time controls); the facilitating effect of prucalopride was not enhanced but significantly decreased in the presence of 10 μM EHNA to a value of 121 ± 11% (*P* < 0.05 versus strips only receiving prucalopride; **Figure 9B**). Vinpocetine, 3 μM, did not influence the EFS-induced submaximal cholinergic contractions (**Figure 9C**), but 10 μM significantly increased them to 130 ± 19% (*P* < 0.05 versus parallel time control strips; **Figure 9E**). The facilitating effect of 0.003 μM prucalopride on EFS-induced contractions (to 165 ± 13%, *P* < 0.001, in the series with 3 μM vinpocetine, and to 146 ± 10%, *P* < 0.01, in the series with 10 μM vinpocetine) was not influenced by 3 or 10 μM vinpocetine (**Figures 9D,F**). Cilostamide, 0.01 μM, did not influence the EFS-induced submaximal cholinergic contractions (**Figure 10A**) but 1 μM reduced them to 74 ± 6% (*P* < 0.05 versus parallel strips going to receive prucalopride in the absence of cilostamide; **Figure 10C**). Neither concentration of cilostamide influenced the facilitating effect of 0.003 μM prucalopride on the EFS-induced contractions (to 140 ± 10%, *P* < 0.01, in the series with 0.01 μM cilostamide, **Figure 10B**; to 132 ± 8%, *P* < 0.01, in the series with 1 μM cilostamide, *P* < 0.01, **Figure 10D**). Rolipram in a concentration of 1 and 3 μM did not influence the EFS-induced submaximal cholinergic contractions (**Figures 10E,G**). Also rolipram did not enhance the facilitating effect of 0.003 μM prucalopride on the EFS-induced contractions (to 140 ± 10%, *P* < 0.01, in the series with 1 μM rolipram, **Figure 10F**; to 143 ± 8%, *P* < 0.001, in the series with 3 μM rolipram, **Figure 10H**).

The results for the jejunum are shown in Supplementary Figures 1, 2. Prucalopride, 0.003 μM, significantly enhanced the EFS-induced submaximal cholinergic contractions in all series. None of the PDE inhibitors enhanced the facilitating effect of prucalopride. The last EFS-induced contraction in the presence of 1 μM cilostamide and prucalopride was significantly higher than that in the presence of prucalopride alone (Supplementary Figure 2D). However, 1 μM cilostamide *per se* reduced EFS-induced contractions by 80% (Supplementary Figure 2C); a mild increase of these small contractions by prucalopride thus already induces a pronounced percentual increase when expressing results as percentage of the mean of the five contractions before adding prucalopride. The results for the colon are given in Supplementary Figures 3, 4. The facilitating effect of 0.003 μM prucalopride on EFS-induced contractions was significantly enhanced by 1 μM cilostamide (Supplementary Figure 4D) and 3 μM rolipram (Supplementary Figure 4H). But again, these concentrations of PDE inhibitor *per se* decreased EFS-induced contractions in a pronounced way (by 84% for 1 μM cilostamide, Supplementary Figure 4C and by 34% for 3 μM rolipram, Supplementary Figure 4G). Mild to moderate facilitation by prucalopride of these small contractions leads to a pronounced increase when expressing results as percentage of the mean of the five contractions before adding prucalopride, but from the expression as percentage of the mean of five contractions, before adding the PDE inhibitor, it is clear that EFS-induced contractions in the presence of 1 μM cilostamide plus prucalopride (Supplementary Figure 4C) or 3 μM rolipram plus prucalopride (Supplementary Figure 4G) are smaller than those in the presence of prucalopride so that no clear-cut evidence for synergy is obtained.

## Discussion

The aim of this study was to investigate whether the intraneuronal pathway of 5-HT_4_ receptors, enhancing cholinergic neurotransmission towards the circular muscle layer of murine gastric fundus, jejunum, and colon is regulated by PDEs and possibly more particular by PDE4, in view of its role in the signal transduction pathway of facilitating 5-HT_4_ receptors in circular muscle of porcine stomach and colon, and human large intestine (Priem et al., 2012, 2013; Lefebvre et al., 2016; Pauwelyn et al., 2018). 5-HT_4_ receptors signal via cAMP; the classic PDEs 1, 2, and 3 are able to metabolize cAMP as well as cGMP, while PDE4 is cAMP specific (Maurice et al., 2003; Lugnier, 2006). These PDEs were therefore assessed by use of selective inhibitors, in addition to the non-selective PDE inhibitor IBMX. It would be optimal to study the influence of PDE inhibition on basal and 5-HT_4_ receptor-stimulated acetylcholine release by measuring acetylcholine release directly. But a set of preliminary experiments showed that our method, applied to measure tritiated acetylcholine release in porcine and human GI tissue (Lefebvre et al., 2016; Pauwelyn et al., 2018), was not able to pick up consistent values in murine GI tissues. When studying then the influence of PDE inhibitors in a functional model where electrically induced cholinergic muscle contractions are enhanced with a 5-HT_4_ receptor agonist, one has to take in account their possible influence at the muscular level. If a given PDE is involved in the breakdown of cAMP (and/or of cGMP for PDE1, 2, and 3) in the murine GI smooth muscle cells, the corresponding inhibitor will induce relaxation by the increase of cAMP and/or cGMP as shown before in the GI tract of other species (Tomkinson and Raeburn, 1996; Jones et al., 2002). The influence of IBMX and the selective PDE1, 2, 3, and 4 inhibitors was therefore first investigated on submaximal cholinergic activity, induced by exogenous carbachol or by endogenous acetylcholine released through electrical field stimulation.

### Influence of PDE Inhibitors on Carbachol- and EFS-Induced Submaximal Contractions

In the three GI regions, the concentration range of IBMX (1–30 μM) inhibiting carbachol-induced submaximal contractions is in agreement with the IC_50_ values of IBMX reported for PDE1-4 (**Table 2**) suggesting the involvement of one or more PDE subtypes in the regulation of murine GI smooth muscle cell activity. To identify the PDE subtype(s) involved, the selective PDE inhibitors were investigated. The selective PDE3 inhibitor cilostamide and the selective PDE4 inhibitor rolipram concentration-dependently decreased the contractions over a large concentration range, the maximal decrease with rolipram being smaller than with cilostamide in the three GI regions. This suggests a role of PDE3 and PDE4 in the regulation of cyclic nucleotide content, with predominance of PDE3. As in contrast to PDE4, PDE3 is also able to metabolize to some extent cGMP in addition to cAMP (Maurice et al., 2003; Lugnier, 2006), some increase in cGMP content might also play a role in the more pronounced inhibitory effect of cilostamide on carbachol-induced contractions than with rolipram. The PDE2 inhibitor EHNA and the PDE1 inhibitor vinpocetine only decreased carbachol-induced contractions in the highest or 2 highest concentrations suggesting either a very minor involvement of PDE1 and/or 2 in the control of muscular cyclic nucleotide content or a non-specific action at PDE3 and/or 4.

Similar effects were observed with the PDE inhibitors when tested on EFS-induced submaximal cholinergic contractions. However, in fundus and jejunum, but not in the colon, cilostamide and rolipram reduced EFS-induced contractions less extensively than carbachol-induced contractions; in the fundus rolipram even did not significantly decrease EFS-induced contractions. While carbachol-induced contraction is a purely postsynaptic phenomenon, presynaptic release of acetylcholine and postsynaptic acetylcholine-induced contraction are involved in EFS-induced contraction so that presynaptic influences of PDE inhibitors can play a role. cAMP-mediated facilitation of acetylcholine release was shown in equine airway cholinergic neurons (Zhang et al., 1996) as well as in guinea pig small intestine (Reese and Cooper, 1984; Yau et al., 1987), where increasing the amount of cAMP by adenylyl cyclase activation with forskolin or PDE inhibition with IBMX, as well as adding cAMP analogs stimulates the release of acetylcholine. If such a presynaptic effect of cilostamide and rolipram occurs in the electrically activated cholinergic neurons of murine fundus and jejunum, the resulting increase in acetylcholine release can be expected to counteract the postsynaptic relaxing effect; this could explain the more pronounced relaxing effects of cilostamide and rolipram on carbachol-induced contractions where presynaptic effects of PDE inhibitors cannot be relevant.

The observation that PDE3 is the main functionally important cyclic nucleotide degrading PDE in murine GI smooth muscle, with a supportive role for PDE4 is in agreement with our previous observations in porcine stomach where PDE3 and 4 have a redundant role, PDE3 being predominant (Priem et al., 2012) and in porcine colon descendens, where PDE3 even seems the sole regulator (Priem et al., 2013). But it contrasts to canine colonic smooth muscle, where PDE4 is functionally the most important PDE in the regulation of smooth muscle contraction with a less pronounced role of PDE3 (Barnette et al., 1993) and the guinea-pig and rat ileum, where next to PDE3 and 4, also PDE5 and possibly PDE1 modulate GI smooth muscle contractility (Tomkinson and Raeburn, 1996).

### Influence of PDE Inhibitors on the Effect of Prucalopride on EFS-Induced Submaximal Cholinergic Contractions

In order to be able to observe a possible facilitating influence of a PDE inhibitor on the facilitating effect of prucalopride, a submaximal concentration of the selective 5-HT_4_ receptor agonist prucalopride was used. In our previous study in the murine GI tract (Pauwelyn and Lefebvre, 2017), cumulative concentration-response curves showed that 0.003 μM prucalopride produced less than 50% of the maximal facilitation of electrically induced submaximal cholinergic contractions. In the actual study, 0.003 μM prucalopride systematically enhanced EFS-induced submaximal cholinergic contractions in the murine fundus, jejunum, and colon but with a quite variable degree of facilitation from series to series. Interseries variability in the facilitating effect of prucalopride was also seen in our previous study for a 10-fold higher concentration, 0.03 μM: 141–204% in fundus, 130–176% in jejunum, and 124–174% in colon (Pauwelyn and Lefebvre, 2017). The highest degree of facilitation with 0.003 μM prucalopride in the actual study never reached the degree of facilitation observable with 0.03 μM, so that further enhancement of the facilitation was possible.

IBMX did not significantly enhance the facilitating effect of prucalopride, but in the fundus a trend for an increase by IBMX was observed. Also, as IBMX was tested versus prucalopride in concentrations reducing EFS-induced contractions at most by 20% [see the section “Influence of PDE Inhibitors on EFS-Induced Submaximal Contractions (**Figures 4–7** and **Table 1A**)”], there cannot be excluded that IBMX in this concentration (3 μM in fundus, 0.3 μM in jejunum, and 1 μM in colon) is insufficiently inhibiting a particular PDE that might be important in facilitating the effect of prucalopride. Reported IC_50_ values at specific PDEs indeed range from 0.8 to 23 μM (**Table 2**). Selective PDE inhibitors were therefore also studied; they were in principle tested in the highest concentration that *per se* did not influence EFS-induced contractions to avoid difficulties in interpreting results (when the PDE inhibitor clearly reduces EFS-induced contractions the action of prucalopride on these reduced contractions is not really comparable to the action of prucalopride on more pronounced control contractions). However when this concentration of the PDE inhibitor was low in comparison to reported IC_50_ values, also a higher concentration was tested even if decreasing the EFS-induced contractions in one or more of the GI regions.

The influence of the PDE inhibitors on the EFS-induced contractions upon single administration [see the section “Influence of PDE Inhibitors on the Effect of Prucalopride on EFS-Induced Submaximal Cholinergic Contractions (**Figures 8–10, Table 1B**, and Supplementary Figures 1–4)] was similar to the effect observed with this concentration when part of the cumulatively administered concentration series [see the section “Influence of PDE Inhibitors on EFS-Induced Submaximal Contractions (**Figures 4–7** and **Table 1A**)”] with exception of 10 μM vinpocetine in fundus and colon. In the cumulative series, 10 μM vinpocetine did not influence and decreased the EFS-induced contractions in fundus and colon, respectively, while single administration increased the contractions in fundus, and showed a biphasic effect, increase followed by decrease, in colon; we have no explanation for these effects.

Neither the PDE1 inhibitor vinpocetine (3 and 10 μM) nor the PDE2 inhibitor EHNA (10 μM) enhanced the effect of prucalopride, suggesting that neither PDE1 nor PDE2 is regulating the signal transduction of the facilitating 5-HT_4_ receptors on cholinergic neurons contracting murine GI circular muscle. The PDE3 inhibitor cilostamide at 0.01 μM did not influence EFS-induced contractions in fundus and jejunum but moderately though significantly decreased them in colon; in this concentration that is lower than most reported IC_50_ values, cilostamide did not enhance the effect of prucalopride. When testing 1 μM, cilostamide significantly enhanced the effect of prucalopride in jejunum and colon, but the interpretation of these results is not reliable due to the very pronounced relaxing effect of the PDE inhibitor as such. The enhancement of the effect of prucalopride is apparent, related to prucalopride acting on contractions with very small amplitude in the presence of cilostamide in comparison to the amplitude of contractions in control tissues. The same is true for the results with 3 μM of the PDE4 inhibitor rolipram in the colon. These data in the murine GI tract of C57Bl/6J mice provide thus no evidence for a PDE-mediated control of the signaling pathway of 5-HT_4_ receptors on cholinergic nerves innervating the circular smooth muscle layer. It cannot be excluded that a possible enhancing effect of PDE3 or PDE4 inhibition on the 5-HT_4_ receptor-mediated facilitation of acetylcholine release is counteracted by the relaxing effect at the muscular level. But obviously, in C57Bl/6J murine GI circular muscle, neither cilostamide nor rolipram allows to obtain more pronounced electrically induced cholinergic contractions with prucalopride. This contrasts to the situation in porcine stomach and colon, and human large intestine where the PDE4 inhibitors rolipram and roflumilast enhance the effect of prucalopride on electrically induced cholinergic contractions and/or acetylcholine release (Priem et al., 2012, 2013; Lefebvre et al., 2016; Pauwelyn et al., 2018) and points to important species differences. It cannot be excluded that the investigation of other mouse strains might lead to different results. But the actual study indicates that the murine C57Bl/6J model, where facilitation of GI cholinergic neurotransmission by 5-HT_4_ receptor agonists is documented, is not suitable for *in vivo* testing of the possible synergistic gastroprokinetic effects of a 5-HT_4_ receptor agonist combined with a selective PDE4 inhibitor.

## Conclusion

In agreement with the porcine GI tract, PDE3 is the main regulator of the cyclic nucleotide turnover in fundus, jejunum, and colon circular smooth muscle of C57Bl/6J mice, with contribution of PDE4. Although 5-HT_4_ receptors are present on cholinergic neurons innervating the circular smooth muscle layer in fundus, jejunum, and colon, the facilitation of electrically induced cholinergic contractions by stimulation of these 5-HT_4_ receptors cannot be enhanced by specific PDE inhibition in contrast to the porcine GI tract. This means that the murine C57Bl/6J model is unsuitable for *in vivo* testing of the possible synergistic gastroprokinetic effects of a 5-HT_4_ receptor agonist combined with a selective PDE inhibitor.

## Ethics Statement

This study was carried out in accordance with the recommendations of the most recent national legislation (Belgian Royal Decree, 29/05/2013) and European Directive (Directive 2010/63/EU). The protocol was approved by the Ethical Committee for Animal Experiments from the Faculty of Medicine and Health Sciences at Ghent University (ECD 14/22).

## Author Contributions

RL designed the study. VP performed the experiments and data analysis. Both interpreted the findings and wrote the manuscript.

## Conflict of Interest Statement

The authors declare that the research was conducted in the absence of any commercial or financial relationships that could be construed as a potential conflict of interest. The reviewer MF and handling Editor declared their shared affiliation.

## Abbreviations

5-HT_4_ receptor: 5-hydroxytryptamine 4 receptor
EFS: electrical field stimulation
EHNA: erythro-9-(2-hydroxy-3-nonyl)adenine
GI: gastrointestinal
IBMX: 3-isobutyl-1-methylxanthine
L-NAME: N_ω_-nitro-L-arginine methyl ester hydrochloride
ns: not significant
PDE: phosphodiesterase
