# Supplementary material for: cAMP Catalyzing Phosphodiesterases Control Cholinergic Muscular Activity But Their Inhibition Does Not Enhance 5-HT4 Receptor-Mediated Facilitation of Cholinergic Contractions in the Murine Gastrointestinal Tract
Source: Front Pharmacol. 2018 Mar 8;9:171. doi: 10.3389/fphar.2018.00171 (PMC5852062; doi:10.3389/fphar.2018.00171)
Supplement: Supplementary file 1 [file Image_1.pdf]

Supplementary Material

cAMP Catalyzing Phosphodiesterases Control Cholinergic Muscular Activity but Their Inhibition Does Not Enhance 5-HT_4 Receptor-Mediated Facilitation of Cholinergic Contractions in the Murine Gastrointestinal Tract

Vicky Pauwelyn and Romain A. Lefebvre*

Department of Pharmacology, Heymans Institute, Ghent University, Ghent, Belgium

*** Correspondence:**
Romain A. Lefebvre
Romain.Lefebvre@UGent.be

1 Supplementary Data

No supplementary Data included.

2 Supplementary Figures and Tables

Supplementary Figures 1 to 4.

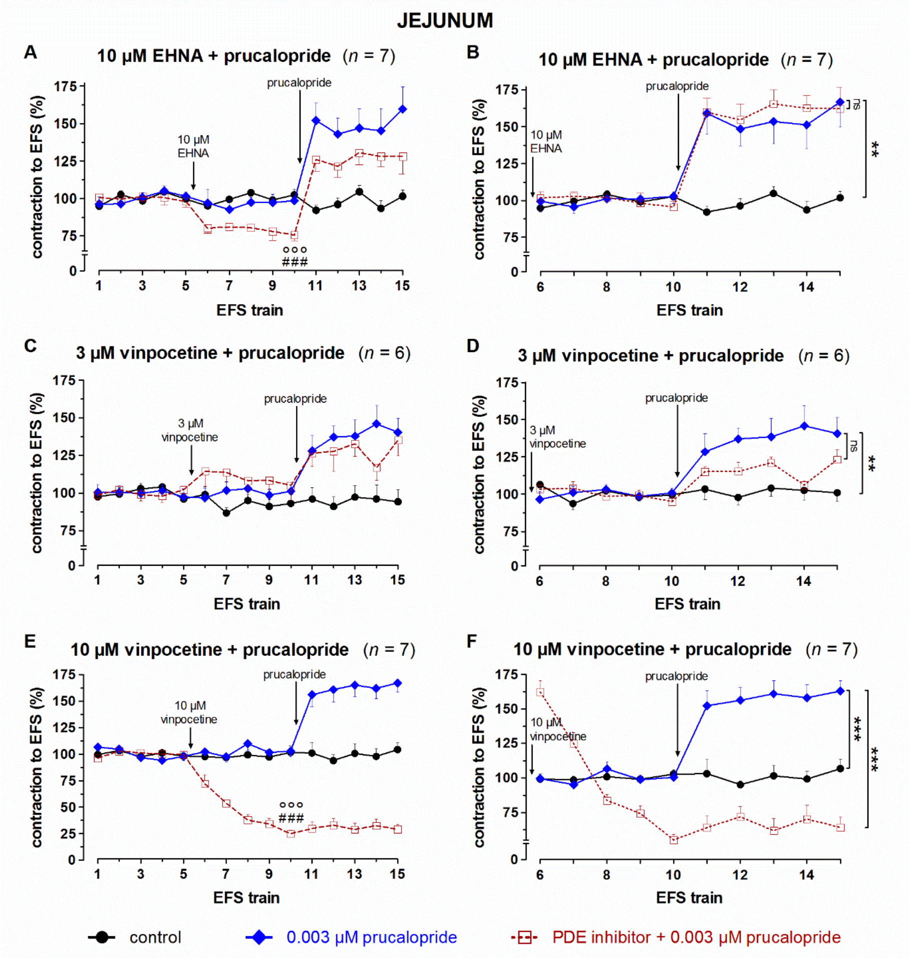

SUPPLEMENTARY FIGURE 1 | Influence of 10 µM EHNA **(A,B)**, 3 **(C,D)** and 10 **(E,F)** µM vinpocetine on the facilitating effect of 0.003 µM prucalopride on submaximal electrically induced cholinergic contractions at $V_{50\%}$ (10 s trains at 8 Hz, 0.5 ms, interval of 10 min) in murine jejunum circular smooth muscle strips. The number of the consecutive stimulation trains is given on the y-axis. Contractions are expressed as percentage of the mean of the five contractions before addition of the PDE inhibitor **(**trains 1-5; **A,C,E)** or of the five contractions in the presence of the PDE inhibitor just before adding prucalopride **(**trains 6-10; **B,D,F)**. Experiments were performed in the continuous presence of 4 µM guanethidine and 300 µM L-NAME. Means ± SEM. Left panels, last EFS-induced contraction before adding prucalopride: °°° $P < 0.001$ versus control and ### $P < 0.001$ versus prucalopride (one-way ANOVA with Bonferroni corrected *t*-test for 2 comparisons i.e., PDE inhibitor versus control and versus prucalopride). Right panels, last EFS-induced contraction: ns not significant, ** $P < 0.01$, *** $P < 0.001$ (one-way ANOVA with Bonferroni corrected *t*-test for 2 comparisons i.e., prucalopride versus control and versus prucalopride in the presence of the PDE inhibitor).

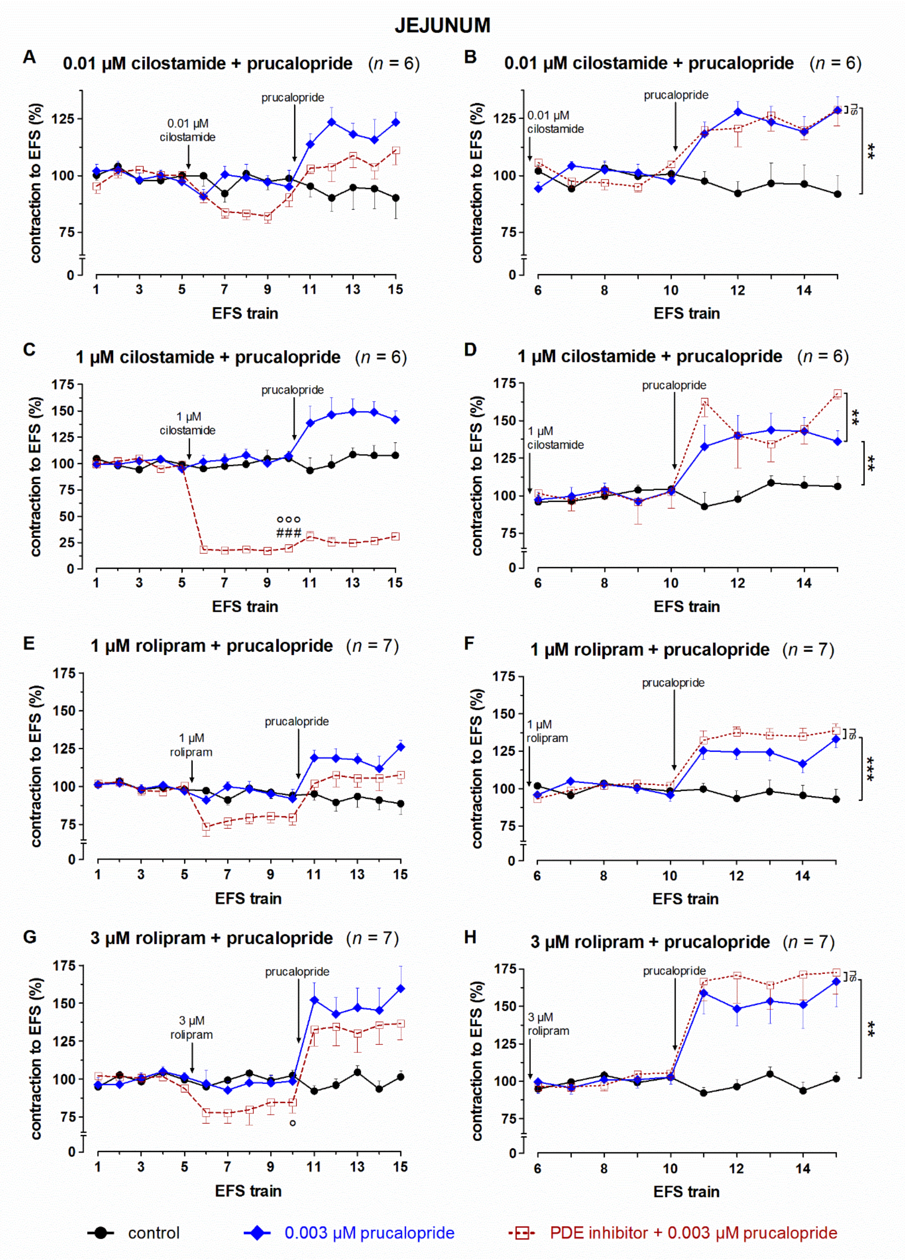

SUPPLEMENTARY FIGURE 2 | Influence of 0.01 **(A,B)** and 1 **(C,D)** µM cilostamide, 1 **(E,F)** and 3 **(G,H)** µM rolipram on the facilitating effect of 0.003 µM prucalopride on submaximal electrically induced cholinergic contractions at $V_{50\%}$ (10 s trains at 8 Hz, 0.5 ms, interval of 10 min) in murine jejunum circular smooth muscle strips. The number of the consecutive stimulation trains is given on the y-axis. Contractions are expressed as percentage of the mean of the five contractions before addition of the PDE inhibitor **(**trains 1-5; **A,C,E,G)** or of the five contractions in the presence of the PDE inhibitor just before adding prucalopride **(**trains 6-10; **B,D,F,H)**. Experiments were performed in the continuous presence of 4 µM guanethidine and 300 µM L-NAME. Means ± SEM. Left panels, last EFS-induced contraction before adding prucalopride: ° $P < 0.05$, °°° $P < 0.001$ versus control and ### $P < 0.001$ versus prucalopride (one-way ANOVA with Bonferroni corrected *t*-test for 2 comparisons i.e., PDE inhibitor versus control and versus prucalopride). Right panels, last EFS-induced contraction: ns not significant, ** $P < 0.01$, *** $P < 0.001$ (one-way ANOVA with Bonferroni corrected *t*-test for 2 comparisons i.e., prucalopride versus control and versus prucalopride in the presence of the PDE inhibitor).

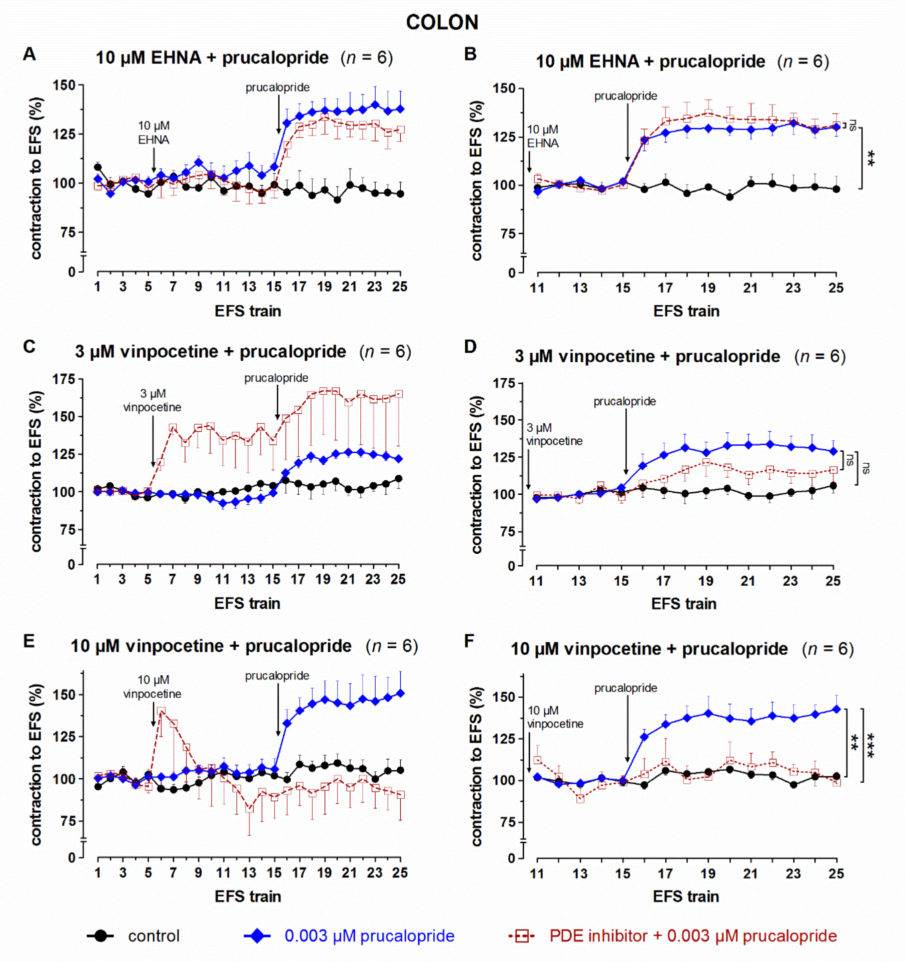

SUPPLEMENTARY FIGURE 3 | Influence of 10 µM EHNA **(A,B)**, 3 **(C,D)** and 10 **(E,F)** µM vinpocetine on the facilitating effect of 0.003 µM prucalopride on submaximal electrically induced cholinergic contractions at $V_{50\%}$ (10 s trains at 8 Hz, 0.5 ms, interval of 5 min) in murine colon circular smooth muscle strips. The number of the consecutive stimulation trains is given on the y-axis. Contractions are expressed as percentage of the mean of the five contractions before addition of the PDE inhibitor **(**trains 1-5; **A,C,E)** or of the five contractions in the presence of the PDE inhibitor just before adding prucalopride **(**trains 11-15; **B,D,F)**. Experiments were performed in the continuous presence of 4 µM guanethidine, 300 µM L-NAME, and 1 µM MRS 2500. Means ± SEM. Left panels, last EFS-induced contraction before adding prucalopride: one-way ANOVA with Bonferroni corrected *t*-test for 2 comparisons i.e., PDE inhibitor versus control and versus prucalopride did not reach significance. Right panels, last EFS-induced contraction: ns not significant, ** $P < 0.01$, *** $P < 0.001$ (one-way ANOVA with Bonferroni corrected *t*-test for 2 comparisons i.e., prucalopride versus control and versus prucalopride in the presence of the PDE inhibitor).

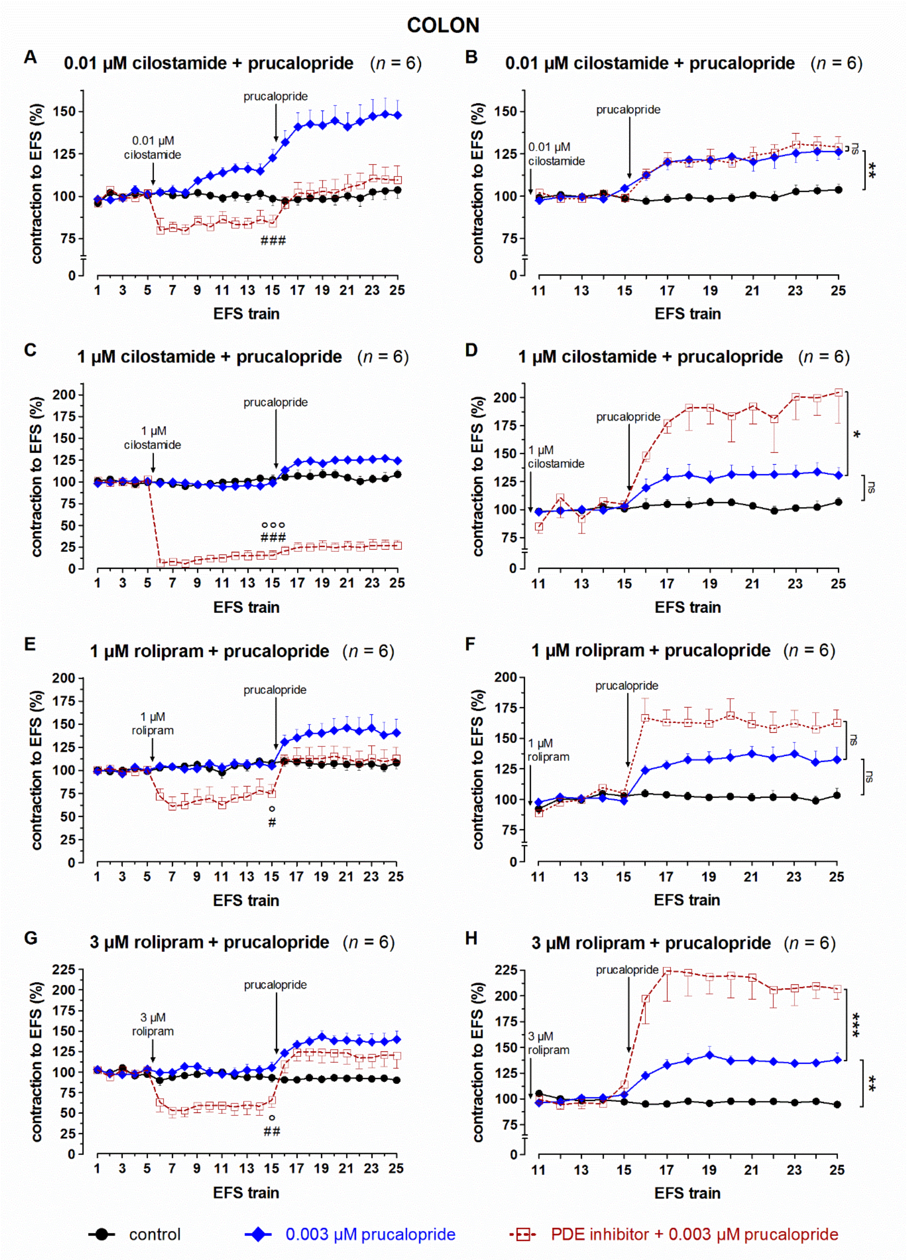

SUPPLEMENTARY FIGURE 4 | Influence of 0.01 **(A,B)** and 1 **(C,D)** µM cilostamide, 1 **(E,F)** and 3 **(G,H)** µM rolipram on the facilitating effect of 0.003 µM prucalopride on submaximal electrically induced cholinergic contractions at $V_{50\%}$ (10 s trains at 8 Hz, 0.5 ms, interval of 5 min) in murine colon circular smooth muscle strips. The number of the consecutive stimulation trains is given on the y-axis. Contractions are expressed as percentage of the mean of the five contractions before addition of the PDE inhibitor **(**trains 1-5; **A,C,E,G)** or of the five contractions in the presence of the PDE inhibitor just before adding prucalopride **(**trains 11-15; **B,D,F,H)**. Experiments were performed in the continuous presence of 4 µM guanethidine, 300 µM L-NAME, and 1 µM MRS 2500. Means ± SEM. Left panels, last EFS-induced contraction before adding prucalopride: ° $P < 0.05$, °°° $P < 0.001$ versus control and $^{\#}$ $P < 0.05$, $^{\#\#}$ $P < 0.01$, $^{\#\#\#}$ $P < 0.001$ versus prucalopride (one-way ANOVA with Bonferroni corrected *t*-test for 2 comparisons i.e., PDE inhibitor versus control and versus prucalopride). Right panels, last EFS-induced contraction: ns not significant, * $P < 0.05$, ** $P < 0.01$, *** $P < 0.001$ (one-way ANOVA with Bonferroni corrected *t*-test for 2 comparisons i.e., prucalopride versus control and versus prucalopride in the presence of the PDE inhibitor).
